# Is *Caretta Caretta* a Carrier of Antibiotic Resistance in the Mediterranean Sea?

**DOI:** 10.3390/antibiotics9030116

**Published:** 2020-03-10

**Authors:** Rosa Alduina, Delia Gambino, Alessandro Presentato, Antonino Gentile, Arianna Sucato, Dario Savoca, Serena Filippello, Giulia Visconti, Giulia Caracappa, Domenico Vicari, Marco Arculeo

**Affiliations:** 1 Department of Biological, Chemical and Pharmaceutical Sciences and Technologies (STEBICEF), University of Palermo, 90028 Palermo, Italy; alessandro.presentato@unipa.it (A.P.); sucato.arianna@gmail.com (A.S.); dario.savoca@unipa.it (D.S.); serenafilippello@gmail.com (S.F.);; 2 Istituto Zooprofilattico Sperimentale della Sicilia “A. Mirri”, 90129 Area Territoriale Palermo, Italy; antogentile1980@gmail.com (A.G.); domenico.vicari@izssicilia.it (D.V.); 3 Area Marina Protetta Isole Pelagie-Comune di Lampedusa e Linosa, 92031 Lampedusa, Italy; giulia.visconti@gmail.com; 4 Centro Recupero Regionale Fauna Selvatica Bosco di Ficuzza, Ficuzza di Corleone, 90034 Palermo, Italy; giulia.caracappa@gmail.com

**Keywords:** antibiotic resistance, antimicrobials, mobile element, heavy metal resistance, loggerhead sea turtle, *caretta caretta*, Mediterranean Sea

## Abstract

Sea turtles can be considered a sentinel species for monitoring the health of marine ecosystems, acting, at the same time, as a carrier of microorganisms. Indeed, sea turtles can acquire the microbiota from their reproductive sites and feeding, contributing to the diffusion of antibiotic-resistant strains to uncontaminated environments. This study aims to unveil the presence of antibiotic-resistant bacteria in (i) loggerhead sea turtles stranded along the coast of Sicily (Mediterranean Sea), (ii) unhatched and/or hatched eggs, (iii) sand from the turtles’ nest and (iv) seawater. Forty-four bacterial strains were isolated and identified by conventional biochemical tests and 16S rDNA sequencing. The Gram-negative *Aeromonas* and *Vibrio* species were mainly found in sea turtles and seawater samples, respectively. Conversely, the Gram-positive *Bacillus*, *Streptococcus*, and *Staphylococcus* strains were mostly isolated from eggs and sand. The antimicrobial resistance profile of the isolates revealed that these strains were resistant to cefazolin (95.5%), streptomycin (43.2%), colistin and amoxicillin/clavulanic acid (34.1%). Moreover, metagenome analysis unveiled the presence of both antibiotic and heavy metal resistance genes, as well as the mobile element class 1 integron at an alarming percentage rate. Our results suggest that *Caretta caretta* could be considered a carrier of antibiotic-resistant genes.

## 1. Introduction

The spread of antimicrobial resistance (AMR) is the main cause of infectious disease treatment failure worldwide, gaining global attention from both an environmental and human health perspective [1]. AMR may be due to either genetic mutations or horizontal transfer of resistance genes, even among non-phylogenetically related bacteria. The acquisition of the antibiotic resistance genes (ARGs) can be considered a natural phenomenon as a result of evolution, although it can be quickened by human influence [2]. Indeed, AMR can be seen as a direct consequence of the misuse of antibiotics, in healthcare, veterinary, agriculture and aquaculture, inexorably leading to the acquirement of antibiotic-resistant traits by microorganisms [3]. Wastewater from agriculture, hospitals, farms or urban wastewater treatment plants could contain resistant bacteria, which, due to either seepage phenomena or sewage from fertilized soils, can contribute to contaminating environments, where ARGs can be transferred to environmental bacteria through the well-known horizontal gene transfer mechanisms [4,5,6]. Indeed, ARGs are frequently associated with gene cassettes containing the class 1 integron and heavy metal resistant genes (HMRGs) [7,8]. Class 1 integron is a potentially mobile genetic element, commonly found in Gram-negative bacteria, responsible for the conjugative-mediated gene transfer [9]. Thus, in both marine and terrestrial environments, wildlife can come into direct contact with most antibiotics, even at sub-inhibitory concentrations [10], leading to the selection of antibiotic-resistant microbiota. In this context, wildlife represents a potential reservoir, or vector, of resistant pathogens and ARGs [1,11,12]; therefore, in the study of the spread of the AMR, it may be useful to evaluate the role of migratory wildlife species, which can carry a resistant microbiota.

Sea turtles are considered an excellent bio-indicator of marine pollution [13,14,15,16,17,18]. Due to their feeding, reproductive habits, longevity and frequency in the coastal areas affected by anthropogenic activities, they could encounter and acquire autochthonous antibiotic-resistant microorganisms of polluted areas.

*Caretta caretta* is the most abundant sea turtle species in the Mediterranean Sea and is the subject of numerous studies and research programs aimed at its protection and conservation. Many events, such as incidental catches by fishing [19,20], water pollution [16], presence of fungi in egg nests [21] and global climatic changes, affect the health status and conservation of sea turtles [22]. To the best of our knowledge, few studies [13,17,18,23] evaluated the frequency of antibiotic-resistant bacteria isolated from adult loggerhead sea turtles in the Mediterranean Sea. Here, the purpose of the study was to evaluate the antibiotic resistance profile of bacteria isolated from loggerhead sea turtles stranded along the coast of Sicily, hatched and/or not hatched egg, sand and seawater, by combining microbiological assay and molecular analysis for detection of antibiotic and heavy metal resistance genes.

## 2. Results

### 2.1. Bacterial Identification

**Figure 1:**
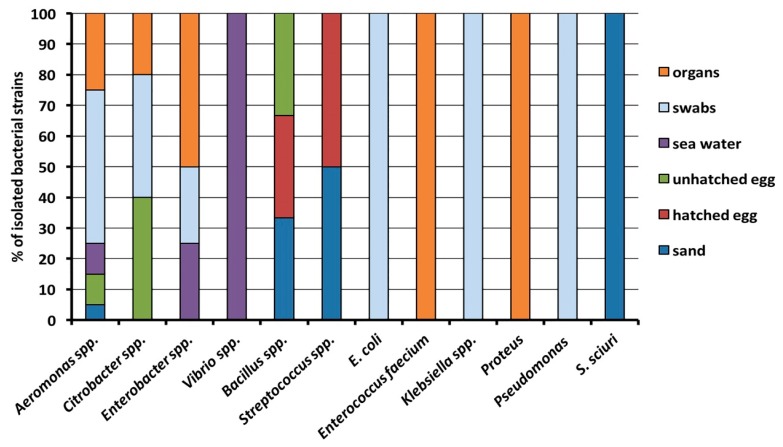
Percentage distribution of bacterial genera in organs, swabs, seawater, eggs and sand.

Among all the samples collected, a total of 44 bacterial strains were isolated and identified through biochemical-enzymatic tests, belonging to 12 different genera between Gram-negative (n = 8) and -positive (n = 4) bacteria. In general, the most abundant isolates belonged to the following genera: *Aeromonas* spp. (n = 20), *Citrobacter* spp. (n = 5), *Enterobacter* spp. (n = 4), *Vibrio* spp. (n = 4), *Bacillus* spp. (n = 3), *Streptococcus* spp. (n = 2); for other genera, only a single strain was isolated (*Escherichia coli*, *Klebsiella* spp., *Staphylococcus sciuri*, *Enterococcus faecium*, *Proteus vulgaris*, and *Pseudomonas* spp.). Particularly, the most ubiquitous bacterial strains were found to be those belonging to *Aeromonas* genera, as they were isolated from each sample category, predominantly found in the different types of swab samples collected (Figure 1). *Citrobacter* bacteria were isolated from the unhatched egg (both yolk and internal eggshell), from an oral and a cloacal swab of two different live sea turtles, as well as from the intestine of a dead one. *Enterobacter* spp. strains were found in a sample of seawater, in a cloacal swab and in two organs of two dead sea turtles. On the other hand, microorganisms belonging to *Bacillus* and *Streptococcus* genera were isolated from samples collected from sand and eggs. *Escherichia coli*, *Klebsiella* spp. and *Pseudomonas* spp. were isolated from a cloacal, skin and an oral swab of live sea turtles, respectively, while *Enterococcus faecium* and *Proteus vulgaris* were obtained from organs. *Staphylococcus sciuri* and *Vibrio* spp. isolates were found only in one sand sample and in the two different seawater ones, respectively.

### 2.2. Antibacterial Resistance

**Figure 2:**
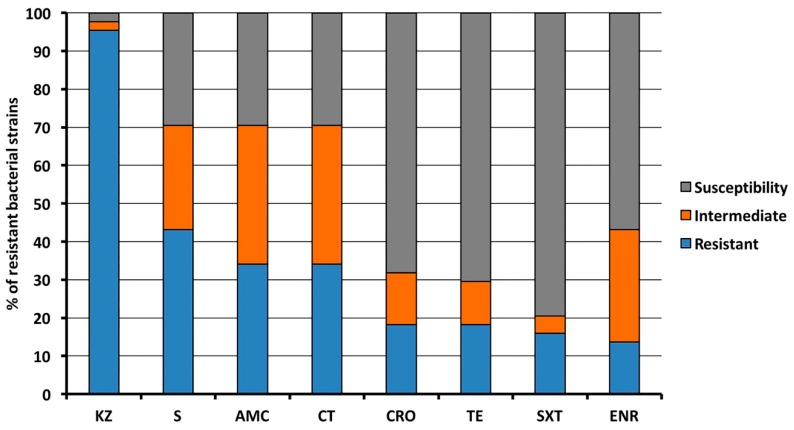
Percentage of isolates resistant, intermediate or sensitive to antimicrobial agents. KZ, Cefazolin; S, Streptomycin; AMC, Amoxicillin/Clavulanic acid; CT, Colistin; CRO, Ceftriaxone; SXT, Sulfamethoxazole/Trimethoprim; TE, Tetracycline; ENR Enrofloxacin.

The antibiotic resistance assay demonstrated that almost all the microbial isolates (exception made for an intermediate and a susceptible isolate from seawater) were resistant to cefazolin (95.5%), while 43.2% of isolates displayed resistance to streptomycin. Furthermore, in decreasing percentage order the isolates were resistant to amoxicillin/clavulanic acid and colistin (both 34.1%), ceftriaxone and tetracycline (both 18.2%), sulfamethoxazole/trimethoprim (15.9%) and enrofloxacin (13.6%). Moreover, high percentages of bacteria displaying an intermediate resistance to amoxicillin/clavulanic acid and colistin (both 36.4%), enrofloxacin (29.5%) and streptomycin (27.3%) were found (Figure 2).

**Figure 3:**
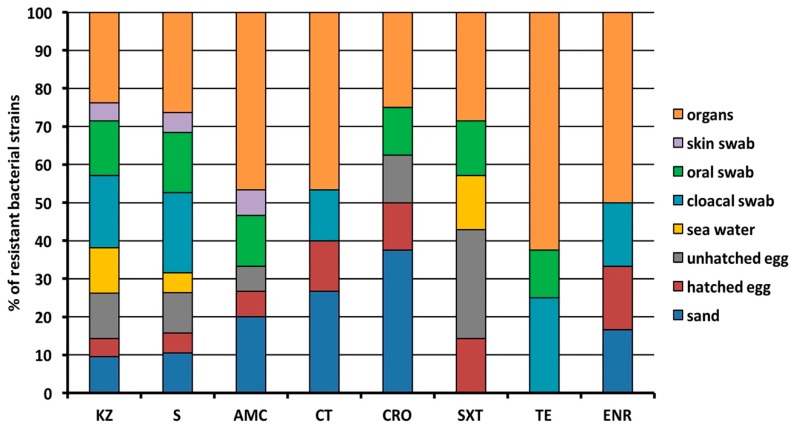
Percentage distribution of resistant isolates from different origin. KZ, Cefazolin; S, Streptomycin; AMC, Amoxicillin/Clavulanic acid; CT, Colistin; CRO, Ceftriaxone; SXT, Sulfamethoxazole/Trimethoprim; TE, Tetracycline; ENR Enrofloxacin.

Organs of the dead sea turtles contained bacteria resistant to all antibiotics tested, as well as bacterial species derived from the hatched egg, with tetracycline being the only exception. A similar trend was also observed for those bacterial strains isolated from sand samples, although sulfamethoxazole/trimethoprim represented an exception along with tetracycline. The oral swab, the unhatched egg together with the cloacal swabs contained antibiotic-resistant isolates against six and five antibiotics, respectively. Finally, seawater samples contained bacteria resistant to cefazolin, streptomycin and sulfamethoxazole/trimethoprim, while skin swabs contained bacteria resistant to cefazolin, streptomycin and amoxicillin/clavulanic acid (Figure 3).

**Figure 4:**
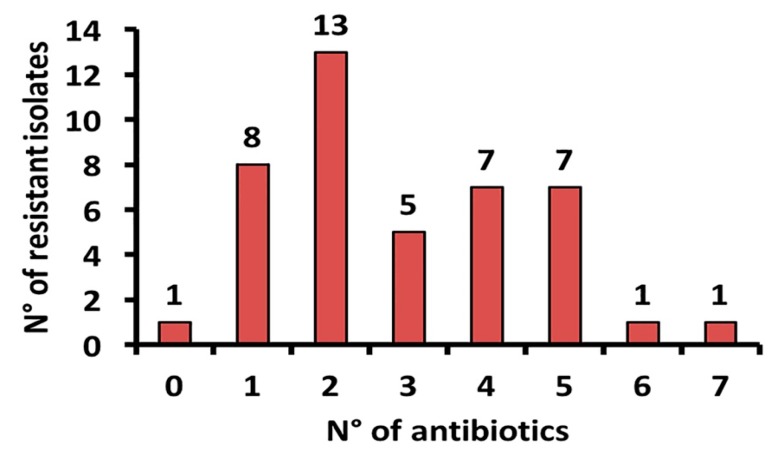
Number of multiple resistant bacteria to antibiotics.

This analysis showed that 13 bacterial isolates were resistant to two out of seven antibiotics tested. On the other hand, those isolates resistant to three, four and five antibiotics were significantly lower, namely, five, seven and seven species, respectively, Notably, only one bacterial strain was resistant to the challenge exerted by either six or seven antibiotics (Figure 4).

### 2.3. Detection of Antibiotic and Heavy Metal Resistance Genes

Metagenomic DNA was extracted from 16 randomly chosen samples and analyzed by PCR for *bla_TEM_*, *bla_CTXM_*, *qnrS*, *sulII* and *tetA* genes, which are the most frequent antibiotic-resistant determinants (Table 1). Moreover, the presence of the mobile element *int1*, as well as *czcA* and *arsB* heavy metal genes were investigated. Almost all samples (81.25%) were positive for the presence of the *bla_TEM_* gene, responsible for beta-lactam resistance, while a lower percentage (31.25%) of metagenomes were positive for sulfonamide resistance gene (*sulII*), and only a few samples (n = 2), derived from live turtles, resulted to be positive for quinolone (*qnrS*) and tetracycline (*tetA*) resistance ones. No *bla_CTXM_* gene was detected. Regarding the resistance to heavy metals, half of the samples (50%) resulted positive for *czcA* (cadmium, cobalt and zinc resistance) and 12.5% were positive for *arsB* (arsenic resistance) genes (Table 1). Surprisingly, the *int1* gene, encoding the mobile element class 1 integron, was found in 15 out of the 16 metagenomes tested.

## 3. Discussion

In this study, we report the isolation and resistance profile of bacteria from loggerhead sea turtles, seawater, sea turtle eggs and sand from the same nest collected along the coast of the Mediterranean Sea. The antimicrobial resistance among bacterial strains isolated from wild marine species is a topic of main concern since these animals have no history of therapeutic antibiotic exposure. The abuse of antibiotics in both human and veterinary medicine—in the last century—showed to also impact diverse environmental niches, which are constantly described as a source of antibiotic-resistant bacteria, possibly contributing to the spreading of the corresponding genes [24]. A few studies have investigated the presence of resistant bacteria in green turtles [14,15,25,26,27,28,29], with the number of scientific reports being even lower on the role of loggerhead sea turtles correlated to emphasized emergence of antibiotic-resistant microorganisms [13,17,18,23,25]. Our results confirm that Gram-negative bacteria are most frequently found in samples deriving from the marine environment, while Gram-positive bacteria are enlaced with an earthly environment [30]. We found *Aeromonas* spp. as a prevalent isolate in all the tested samples, mainly in organs and swabs of sea turtles. *Aeromonas hydrophila* is an emergent bacterial pathogen often isolated in marine and coastal environments [31], and although no statistics can be done for the low number of samples analyzed, we surmised that the prevalence of *Aeromonas* could be due either to different health conditions of the turtles or the environmental contamination condition of the Mediterranean Coast of Sicily. The latter hypothesis is also supported by the presence of *Aeromonas* spp. in unhatched eggs, sand and seawater samples, which is in line with the finding of a previous report [21]. The presence of *Citrobacter* spp. in our samples was in line with other reports as these environmental bacteria were often isolated from both free-living and captive sea turtles, as well as from swab sampling of different organs [13,17,18,23,25,29].

Bacterial isolates were resistant to the first-generation beta-lactam antibiotic cefazolin (95.5%), result in agreement with the resistance levels (92.6% and 94.7%) reported previously [13,18] for those microorganisms found in both cloacal and oral swabs. Here, similarities also extend to the levels of streptomycin (43%) and trimethoprim-sulfamethoxazole (16%) resistant strains with those reported by the same authors mentioned above [13,18]. Surprisingly, the isolates of this study showed a lower resistance level to tetracycline (18%) and enrofloxacin (13.6%) with respect to previous studies [13,18], overall reporting higher resistance percentages for these antibiotics. Noteworthy, enrofloxacin is a veterinary antibiotic used during therapy in recovery centers and, since enrofloxacin resistant strains were found in sand and hatched eggs, it might suggest that the spreading of these resistant microbes into the marine environment can be augmented by the sea turtles treated with enrofloxacin and released back to the sea. We surmise that sea turtles could acquire both ARGs and antibiotic-resistant bacteria from the contaminated seawater and sand, although rivers, lakes and seawater are environments with a low bacterial density in comparison to soils. However, several studies have noted the resistance burden in aquatic environments, as well [32]. During egg deposition, ARGs and antibiotic-resistant bacteria could be transferred from the mother to the eggs/sand of the nest; therefore, based on this hypothesis, sea turtles could be considered a carrier and spreader of resistance. In line with this, here we noted that organs of dead turtles and sand were the samples with the highest level of antibiotic-resistant strains. Moreover, we correlated the detection of antibiotic-resistant isolates to antibiotic contamination of anthropogenic origin, since not all the isolates of the same species (e.g., *Aeromonas* spp.) were resistant to the same antibiotic. If natural immunity occurred, this should be present in all the isolates of the same species. However, we cannot rule out this aspect.

Multidrug resistance was evidenced mainly in isolates from organs of dead sea turtles; indeed, two isolates displayed resistance to six and seven out of the seven tested antibiotics (Figure 4). Moreover, microbial isolates resistant to five antibiotics were found in both hatched egg and sand, suggesting that antibiotic contamination of anthropic nature could generate a hostile environment for eggs and/or hatched ones, therefore determining that turtles could receive antibiotics or antibiotic resistance genes from the environment, making it an “ideal host” for bacteria to consequently spread. Conversely, the less resistant isolates were found in seawater samples, displaying resistance mainly to one antibiotic, while only one isolate was resistant to two antibiotics and another was sensitive to all antibiotics tested (Figure 4). Reasonably, seawater has a lower bacterial density; thus, this could be the reason why the seawater samples contained less resistant isolates [33]. The resistance to antibiotics found in our samples, as well as in those reported by other authors [13,18], could be the result of the widespread misuse and subsequent release into the environment of antibiotics used in the human and veterinary medical field, in aquaculture and the zootechnical ones, contributing to generate an increase in antibiotic-resistant bacteria found in diverse marine habitats [4,14,28,34]. Moreover, the presence of antibiotic-resistant bacteria in some marine fishes is related to spill events of urban wastewater into the sea [5].

The antibiotic-resistant strains found in eggs and yolk open not very comforting prospects. The possible implications for the health of marine organisms as well as on human health could be potentially devastating. This dramatic picture is even more emphasized by the presence of multi-resistant strains found in the two coastal water samples far from urban waste spills. Several authors have highlighted the presence of resistant bacteria in surfers, which would be acquired by the accidental ingestion of the contaminated seawater [35]. In this context, sea turtles can be considered as “concentrators” of antibiotic-resistant bacteria since they carry a resistant microbiota in a diluted environment. This resistance would make it difficult for veterinarians to treat pharmacologically individuals of stranded sea turtles with serious pathologies. Additionally, the presence of antibiotic-resistant strains found in the nests caused by contact between the oviduct of the mother and the egg (shell and yolk) [28] can depend directly on the seawater, which, in our case, can reach the nests during the incubation period through storm and tidal flow. This means that sea turtles already from the first days of life have an acquired antibiotic-resistant microbiota that could somehow interfere with the natural immune response, facilitating the onset of pathologies. Moreover, these microbes could be diffused by the consumption of sea turtle meat that is still used in some countries [36]. Finally, we must consider that the sea turtles could be predated by sharks and fishes with a worrying probability to spreading antibiotic-resistant strains in the food web. In addition, the diffusion of resistant strains in marine species could be enhanced by the spread of ARGs in various environments. As a result of the use of human and veterinary antibiotics, hospital wastewater and livestock manure are considered the major sources of environmental ARGs [37]. On this matter, the detected ARGs confer resistance against four different groups of antibiotics out of the five tested: tetracyclines (*tetA*), beta-lactamases (*blaTEM*), sulphonamides (*sulI*) and quinolone (*qnrS*) with a quantitative prevalence of *blaTEM*. The presence of ARGs themselves in low impacted areas is not surprising; in fact, ARGs were also found in more remote areas such as Arctic ice [38], as well as in samples attributable to the pre-antibiotic era [39]. The high percentage of the *int1* gene in almost all the samples analyzed is truly worrisome, since the coded mobile element can determine gene transfer, thus exasperating the spread of ARGs in the near future.

Our results contribute to enrich the information reported by previous studies on marine species in which drug-resistant bacteria arising from marine environments were discussed. For example, resistant bacteria were isolated from eggshell layers, albumen and yolk of green turtle (*Chelonia mydas*) eggs [28], and from fecal and blowhole swabs of wild bottlenose dolphins (*Tursiops truncatus*) [12]. Resistant strains were detected in fecal samples of wild South American fur seals (*Arctocephalus australis*) and Subantarctic fur seals (*Arctocephalus tropicalis*) [40], as well as from fecal samples of wild marine species [41]. To the best of our knowledge, this is the first report on resistome of samples from sea turtles, a part from a report on the presence of ampicillin resistance genes found in *Citrobacter sp.* isolated from nesting turtles of the species *C. mydas* [42]. These studies indicate that marine organisms were and are yet exposed to polluted effluents during their migratory routes and feeding. Therefore, bacterial resistance in these animals highlights the impact of human activities in both the environment and the antibiotic resistome [43].

A thorough understanding of both persistence and diffusion of antibiotics, as well as that of different antibiotic-resistant genes into the environment, is far from being accomplished, neither at a local nor at a global scale [44,45]. The understanding of the ecological role, the spread and the persistence of antibiotic resistance, both in terms of antibiotic-resistant strains and ARGs in the environment are crucial steps to develop effective control action. Thus, studies of marine species provide information that can serve to ameliorate protection and conservation measures on these species and the marine environment itself.

## 4. Materials and Methods

### 4.1. Sample Collection

Bacteria were isolated from organs (i.e., lung, heart, intestine, liver, spleen and kidney) of eight dead loggerhead sea turtles, as well as from eight cloacal, six oral and two skin swabs of 14 sea turtles upon their arrival at the Regional Centre for the Recovery of Sea Turtles at the Veterinary Public Health Institute of Sicily (IZSSi), during the years 2018–2019. All the loggerhead sea turtles were conferred to the personnel of the Regional Centre for the Recovery of Sea Turtles at the IZSSi. Morphometric data such as sex, body weight and curved carapace length (CCL) were recorded and listed in Table 2. During hospitalization of live individuals, the sea turtles were housed separately in individual tanks, which were previously cleaned and disinfected with regular bleach, and containing seawater as described elsewhere [46]. Every two days, tanks were cleaned and the water replaced with fresh water. During the recovery period, turtles were fed twice a week with small pelagic fishes. The sample collection was performed within two days of turtles’ arrival at the Centre.

During autopsies of dead sea turtles, the state of the specimen was assessed by specialized veterinaries of the recovery center, with only fresh organs being sampled to avoid undesired microbial contaminations due to the advancement decomposition of turtles’ specimens. Afterwards, organs were transferred to the laboratory in sterile jars. About 1 g of the organ was inoculated in the enrichment broth (9 mL). For plate seeding, an incision was made with sterile scalpels and tweezers and the loop was introduced, which was afterward sowed on specific agar plates.

Furthermore, bacteria were isolated from swabs of the external part, of the yolk, and of fragments deriving from of unhatched eggs, as well as from four samples of sand—two of which were harvested superficially and the remaining ones at 40 cm deep—located nearby the turtles’ nests monitored on the Linosa Island (Mediterranean Sea) in the summer of 2018. Finally, two 1 L samples of seawater were collected along the South and North coast of the Mediterranean Sea using sterilized glass containers. Samples were transferred to the IZSSi in sterile biological bags for laboratory analyses, kept at 4 °C until their processing. For clarity, all the samples investigated in this study are reported in Table 2 and Table 3. Collection of samples was conducted in strict accordance with the recommendations of the Region of Sicily and the Ministry of Health (regional law n. 6067/2013 and national law n. 96/2016 and 0017054.25-07-2018).

### 4.2. Bacterial Isolation

Microbial isolation was performed by seeding each sample onto Columbia Agar with 5% Sheep Blood, as well as onto selective media, namely: (i) Mannitol Salt agar to isolate *Staphylococcus* spp., and (ii) MacConkey agar for *Enterobacteriaceae*, *Pseudomonas* and *Aeromonas* species. The isolation of *Salmonella* spp. was performed by enrichment cultures from 1 g of organ put in 9 mL of alkaline peptone water broth, Selenite Cystine, or Rappaport Vassiliadis broth at 37 °C for 24–48 h at 180 rpm. Afterward, aliquots of cultures were spread onto xylose lysine desoxycholate agar and brilliant green agar, the plates being incubated at 37 °C for 24–48 h under static mode. All the media were purchased from Oxoid. The isolated strains were identified by biochemical-enzymatic tests such as catalase, oxidase, mobility, indole, sugar fermentation, citrate and urea metabolism as described in [21]. When the biochemical analysis was not exhaustive, amplification and sequencing of the 464 bp fragment of the 16S rDNA were carried out. An aliquot (2 µL) of the bacterial lysate, prepared as previously described [47], was used to amplify the 464 bp internal fragment of the 16S rDNA using One Taq DNA polymerase (NEB), using primer pairs and the corresponding annealing temperatures listed in Table 4. After confirmation through agarose (1% *w*/*v*) gel electrophoresis, the polymerase chain reaction (PCR) products were purified and sequenced at Macrogen Inc. (Seoul, Korea, sequencer). The Nucleotide sequences were identified using the NCBI nucleotide BLAST.

### 4.3. Antibiotic Susceptibility Test

The antibiotic susceptibility of the bacterial strains isolated was performed using the Kirby-Bauer method on Muller Hinton agar, by testing their sensitivity to eight antibiotics, namely, amoxicillin/clavulanic acid (AMC, 30 µg), cefazolin (KZ, 30 µg), ceftriaxone (CRO, 30 µg), colistin (CT, 10 µg), streptomycin (S, 10 µg), enrofloxacin (ENR, 5 µg), sulfamethoxazole/trimethoprim (SXT, 25 µg) and tetracycline (TE, 30 µg), as described elsewhere [48]. Interpretation of results was carried out by referring to the Clinical & Laboratory Standards Institute (CLSI) 2018 range. Antimicrobial disks were obtained from Oxoid (United Kingdom).

### 4.4. Detection of Antibiotic and Heavy Metal Resistance Genes

The metagenomic DNA was extracted from samples of three either dead or alive sea turtles, from three fragments of eggshell, from the yolk of two unhatched eggs, three sand samples and two seawater ones using the protocol reported in [46].

Metagenomic DNA was utilized as template to amplify the genes coding for products responsible for the resistance to antimicrobials, such as tetracycline *tetA*, sulfonamides *sulII*, β-lactams *blaTEM* and *blaCTXM* and quinolones *qnrS*. Moreover, the *int1*, *arsS* and *czcA* genes were investigated. All PCR reactions were performed using the annealing temperature and the primer pairs listed in Table 4. The presence of the expected amplification product was considered as a positive sample. As a control, the 142 bp DNA fragment of the 16S rDNA gene was used Table 4.

## Figures and Tables

**Table 1:** Summary of the presence/absence of ARGs, HMRGs, and *int*1 gene in the 16 metagenome samples analyzed.

Sample	*bla_TEM_*	*qnrS*	*sulII*	*tetA*	*bla_CTXM_*	*czcA*	*arsB*	*int1*
Dead Turtle (n = 3)	2	ND ^1^	1	ND	ND	1	ND	2
Live Turtle (n = 3)	2	1	2	1	ND	1	ND	3
Sand (n = 3)	3	ND	ND	ND	ND	3	ND	3
Eggshell (n = 3)	2	ND	1	ND	ND	1	2	3
Yolk (n = 2)	2	ND	ND	ND	ND	ND	ND	1
Seawater (n = 2)	2	ND	1	ND	ND	2	ND	2

^1^ ND indicates that the correct amplification product was not obtained.

**Table 2:** Details of samples.

Sample	Location	Bacterial Isolates
Bottom sand	Linosa	*Staphylococcus aureus*; *Aeromonas hydrophyla/cavie*
Top sand		*Bacillus* sp.; *Streptococcus* sp.
Hatched egg		*Bacillus* sp.; *Streptococcus* sp.
Unhatched egg		*Aeromonas hydrophyla/cavie*; *Citrobacter freundii*; *Bacillus* sp.
Yolk		*Aeromonas hydrophyla/cavie*; *Citrobacter freundii*;
Seawater	South coast	*Enterobacter* sp.; *Vibrio alginolyticus*; *Aeromonas* sp.
	North coast	*Vibrio neocaledonicus*; *Vibrio* sp.

**Table 3:** Details of samples.

Sample	Sex	CCL ^1^	Weight (Kg)	Stranding Location	Isolate
Cloacal swab	F	68	29	Siracusa (SR)	*Aeromonas* spp.
	F	41	18		*Citrobacter* spp.
	F	38	19	Lipari (ME)	*Escherichia coli*
	-	13	4	Augusta (SR)	*Aeromonas* spp.
	-	25	8		*Enterobacter* spp.
	-	30	16		*Aeromonas* spp.
	M	25	11		*Aeromonas* spp.
	-	46	23	Milazzo (ME)	*Aeromonas* spp.
Oral swab	F	54	23	Milazzo (ME)	*Pseudomonas* spp.
					*Citrobacter* spp.
					*Aeromonas* spp.
	F	38	14	Lipari (ME)	*Aeromonas* spp.
	M	54	23	Palermo (PA)	*Aeromonas* spp.
	M	48	26	Filicudi (ME)	*Aeromonas* spp.
Skin swab	F	31	10	Ustica (PA)	*Klebsiella* spp.
	-	28	10	Porticello (PA)	*Aeromonas* spp.
Organs	F	51	34	Milazzo (ME)	*Aeromonas* spp.
					*Enterococcus faecium*
	-	27	4		*Aeromonas* spp.
	-	38	35		*Proteus vulgaris*
	F	68	29	Siracusa (SR)	*Aeromonas* spp.
	F	44	31	Terrasini (PA)	*Aeromonas* spp.
	F	48	21		*Aeromonas* spp.
					*Citrobacter* spp.
	M	43	19	Messina (ME)	*Enterobacter* spp.
	M	23	3.5		*Enterobacter* spp.

**Table 4:** List of primers used in this study.

Target Name	Primer Sequence (5′-3′)	Annealing Temperature	Amplicon Size (bp)	Reference
16SrDNA	CGGTGAATACGTTCYCGG GGHTACCTTGTTACGACTT	55	142	[49]
*tet*A	GCTACATCCTGCTTGCCTTC CATAGATCGCCGTGAAGAGG	64	210	[50]
*bla*_TEM_	TTCCTGTTTTTGCTCACCCAG CTCAAGGATCTTACCGCTGTTG	60	112	[51]
*bla*_CTXM_	CTATGGCACCACCAACGATA ACGGCTTTCTGCCTTAGGTT	60	103	[52]
*qnr*S	GACGTGCTAACTTGCGTGAT TGGCATTGTTGGAAACTTG	62	118	[53]
*sul*II	TCCGGTGGAGGCCGGTATCTGG CGGGAATGCCATCTGCCTTGAG	60	191	[54]
*czc*A	TCGACGGBGCCGTGGTSMTBGTCGAGAA GTVAWSGCCAKCGGVBGGAACA	63	232	[55]
*ars*B	GTSAARCCSTTYTCGATGGC GCRAASGCSAHSAYCATGAT	56.5	226	[55]
*int1*	GGCTTCGTGATGCCTGCTT CATTCCTGGCCGTGGTTCT	59	148	[56]
16S rDNA	CCTACGGGNBGCASCAG GACTACNVGGGTATCTAATCC	55	464	[57]
